# Prevalence and Impact of Antidepressant and Anti-Anxiety Use Among Saudi Medical Students: A National Cross-Sectional Study

**DOI:** 10.3390/healthcare13151854

**Published:** 2025-07-30

**Authors:** Daniyah A. Almarghalani, Kholoud M. Al-Otaibi, Samah Y. Labban, Ahmed Ibrahim Fathelrahman, Noor A. Alzahrani, Reuof Aljuhaiman, Yahya F. Jamous

**Affiliations:** 1 Department of Pharmacology and Toxicology, College of Pharmacy, Taif University, P.O. Box 11099, Taif 21944, Saudi Arabia; 2 Stroke Research Unit, Taif University, P.O. Box 11099, Taif 21944, Saudi Arabia; 3 Department of Chemistry, Faculty of Science, Al-Baha University, P.O. Box 1988, Albaha 65799, Saudi Arabia; khalroqi@bu.edu.sa; 4 Department of Physiology, Faculty of Medicine, Umm Al-Qura University, Makkah 21955, Saudi Arabia; sylabban@uqu.edu.sa; 5 Department of Clinical Pharmacy, College of Pharmacy, Taif University, Taif 21944, Saudi Arabia; aihassan@tu.edu.sa; 6 Department of Biochemistry, Faculty of Sciences, King Abdulaziz University, Jeddah 23218, Saudi Arabia; nsalahalzahrani@stu.kau.edu.sa; 7 Wellness and Preventive Medicine Institute, Health Sector, King Abdulaziz City for Science and Technology, Riyadh 11442, Saudi Arabia; reuof.aljuhaiman@hotmail.com

**Keywords:** antidepressants, anti-anxiety medications, medical students, mental well-being

## Abstract

**Background:** Mental health issues among medical students have gained increasing attention globally, with studies indicating a high prevalence of psychological disorders within this population. The use of antidepressants and anti-anxiety medications has become a common response to these mental health challenges. However, it is crucial to understand the extent of their usage and associated effects on students’ mental health and academic performance. This cross-sectional study explored the use of antidepressants and anti-anxiety drugs and their impact on the mental health of medical students in Saudi Arabia. **Methods:** A cross-sectional survey of 561 medical students from 34 universities was conducted between March and July 2024. An anonymous online questionnaire was used to collect sociodemographic, mental health, and medication usage-related information. **Results:** Most of the participants were female (71.5%) and aged 21–25 years (62.7%). Approximately 23.8% of them used antidepressants, 5.6% reported using anti-anxiety medications, and 14.0% used both types of medication. Among the medication users, 71.7% were using selective serotonin reuptake inhibitors (SSRIs), and 28.3% were using other medications. Adverse drug reactions were reported by 58.8% of the participants, and 39.6% changed drugs with inadequate efficacy. Notably, 49.0% of the respondents who have ever used medications discontinued their medication without consulting a healthcare professional. Despite these challenges, 62.0% of the participants felt that their medications had a positive impact on their academic performance, 73.4% believed that the benefits outweighed the drawbacks, and 76.2% expressed a willingness to continue taking their medication. In particular, 77.6% agreed that treatment with these drugs could prevent mental breakdowns. Sleep duration, physical activity, and family history of psychiatric disorders were significantly associated with medication use, with *p* values of 0.002, 0.014, and 0.042, respectively. **Conclusions:** These results shed light on the need to understand the prescribing practices of antidepressant and anti-anxiety drugs among medical students while promoting the appropriate use of these medications among the students. There is a need to incorporate mental health interventions into counseling services and awareness programs to support students. Future longitudinal studies are needed to explore long-term trends.

## 1. Introduction

Psychological and mental health issues among university students are considered escalating public health problems worldwide, particularly among medical students, since they experience different and more acute stressors than their colleagues in other disciplines do [1,2]. The demanding nature of medical education, characterized by excessive workload, academic pressure, peer pressure, competition, a stressful environment, a lack of sleep, and financial difficulties, contributes to elevated levels of stress, anxiety, and depression [3,4]. Medical students are negatively affected by these pressures, which can indicate poor academic performance, dropping out of school, impaired clinical practice, drug addiction, and increased suicide rates [5]. Depression is the most common psychiatric disorder among medical students; several studies have shown that the prevalence of depression has increased in this population [3,4,6]. A meta-analysis of 77 studies revealed that 28.0% (95% confidence interval [CI 24.2–32.1%] of medical students worldwide suffer from depression, with the incidence varying by age, sex, and year of study [7]. Notably, studies conducted in Saudi Arabia have reported a higher prevalence of depression among medical students, ranging from 30.9% to 77.6%, with an average of approximately 51.5% [8]. This suggests a localized burden that warrants focused investigation within the national context.

Antidepressants are usually prescribed to treat anxiety and depressive disorders and play an important role in controlling mental health issues, improving mood, alleviating depressive symptoms, and improving overall functioning if used under medical supervision and with controlled use [9]. Nevertheless, the prevalence, trends, and impact of antidepressant use on the personal and academic lives of medical students are of great concern. The high academic pressure faced by medical students and the stigma attributed to mental health challenges may discourage students from seeking help and accelerate the onset of psychiatric disorders [10,11]. As a result, some students turn to antidepressants as a coping strategy to address their mental health problems [12]. Studies have shown that antidepressant use is widespread among medical students, indicating a worrying trend [13,14,15]. Administration of antidepressants may entail a risk of a wide range of side effects, which vary in occurrence and intensity, depending on the individual’s sensitivity, medication class, and treatment duration [16]. Commonly reported side effects include drowsiness, anxiety, weight gain, insomnia, dizziness, headache, dry mouth, blurred vision, and nausea, which were all indicated by the patients while on antidepressant therapy [17]. In certain cases, serious, life-threatening consequences may arise, such as serotonin syndrome, increased suicidal thoughts and behavior, particularly among pediatric and young adult patients [18,19]. Notably, a considerable portion of these side effects manifest within the first few weeks of treatment and typically subside with continued use [20].

The uncontrolled and long-term use of antidepressants by medical students can lead to psychological dependence and withdrawal symptoms if discontinued abruptly, which can have a negative impact on their health, academic performance, and future professional roles [21,22]. Other studies show that antidepressant use in this population is significantly associated with mental health problems and deficits in academic performance [23,24,25]. Consequently, the use of antidepressants by medical students is a critical and significant issue since it may have an impact on their performance, health, and ability to concentrate [26].

Despite growing global attention to this issue, there remains a distinct gap in the literature regarding the patterns, determinants, and impact of antidepressant and anti-anxiety medication use among medical students in Saudi Arabia. This study aims to address this gap by investigating the prevalence of antidepressant and anti-anxiety drug use among Saudi medical students, as well as the potential impact on their academic performance and mental health. The findings are expected to provide much-needed insight into local trends and help guide universities and healthcare institutions in designing appropriate awareness programs, guidelines, and curriculum enhancements.

## 2. Methods

### 2.1. Ethical Approval

Ethical approval was acquired from the Umm Al-Qura University review board (HAPO-02-K-012-2024-02-2054). Informed consent was obtained from all participants at the start of the questionnaire, ensuring confidentiality and the right to withdraw from the study at any time.

### 2.2. Study Design and Participants

This analytical cross-sectional study was conducted between mid-March 2024 and July 2024 to examine the usage of antidepressant and anti-anxiety medications and their associations with mental well-being among medical students in Saudi Arabia. Inclusion criteria included all medical students currently enrolled in Saudi universities during the 2023–2024 academic year. Exclusion criteria involved nonmedical students and those who did not provide informed consent.

Convenience sampling was used to ensure that the participants were from several academic years and regions. We sent the questionnaire via the authors’ mailing lists to 34 medical schools, and additional social networking was utilized to contact more colleges. The remaining institutions were reached in an indirect manner through friends, colleagues, and social networks.

### 2.3. Data Collection

The participants completed an online, anonymous questionnaire via Google Forms, each of which took approximately 10 min. The questionnaire link was sent by email to facilitate responses from various students. The exact response rate could not be determined due to the online nature of the survey, and the use of social networks and indirect distribution methods besides emails.

### 2.4. Instruments

The analysis used a validated structured questionnaire to collect sociodemographic data, mental health status, and use of antidepressants and anti-anxiety medications among medical students. The sociodemographic items contained information about participants’ age, sex, academic year, university of study, socioeconomic status, and family history of psychiatric illness. For the assessment of mental health, a questionnaire based on the Diagnostic and Statistical Manual of Mental Disorders, Fifth Edition (DSM-5) was used. Questions focused specifically on symptoms associated with major depressive disorder (MDD) and generalized anxiety disorder (GAD). Participants indicated current medication use for antidepressants and anti-anxiety medications (e.g., duration of use), medication class (e.g., selective serotonin reuptake inhibitors (SSRIs)) and occurrence of side effects. Additionally, the survey investigated the participants’ attitudes toward the impact of these drugs on their performance at school. This comprehensive approach ensured a thorough assessment of both mental health and medication usage among the student population.

### 2.5. Survey Validity and Reliability

A pilot study involving 15 individuals was conducted to ensure the quality and clarity of the survey questions, as well as to determine the appropriate time needed to complete the survey. The results from the pilot participants were not included in the final analysis. The primary goal of the pilot survey was to assess the reliability of the questionnaire, which was evaluated via Cronbach’s alpha.

### 2.6. Statistical Analysis

Statistical analysis was accomplished via SPSS (IBM version 26). Descriptive statistics were employed to describe sociodemographics and mental status. Categorical variables are displayed as frequencies and percentages. The chi-square test was used to determine links between antidepressant and anti-anxiety medication consumption and sociodemographic variables. Logistic and linear regression analyses were conducted to examine the associations between medication use and various predictors.

We determined the *p* value of the Hosmer-Lemeshow chi-square test for logistic regression, as well as the adjusted R-squared value for linear regression. The resulting models produced odds ratios (ORs), standardized beta coefficients, and 95% confidence intervals (CIs). A *p*-value < 0.05 was considered statistically significant.

## 3. Results

### 3.1. Sociodemographic Data

The study included 561 medical students; most were female (71.5%) and aged 21–25 years (62.7%). Most students were from the western region (42.6%) of Saudi Arabia. The participants were from various universities, with the largest group from King Saud bin Abdulaziz University for Health Sciences (Riyadh, 20.1%), followed by Ummul Qura University (Makkah, 19.8%). In terms of academic year, first-year students represented the largest group (22.3%), and the majority reported their academic performance as either “very good” (33.7%) or “excellent” (30.8%). Most students’ mothers had a ‘bachelor’s degree’ (53.7%), as did their fathers (43.3%). The majority of the participants came from middle-class socioeconomic backgrounds (51.9%), and 51.0% reported sleeping between 6 and 8 h per day. The physical activity levels varied, with 41.7% reporting no regular exercise. A significant portion (70.4%) reported no family history of psychiatric disease, and smoking was relatively uncommon, with 95.7% not smoking cigarettes and 94.3% not smoking water-pipe (Shisha) (Table 1).

### 3.2. Understanding Anxiety and Depression Among Medical Students

**Figure 1:**
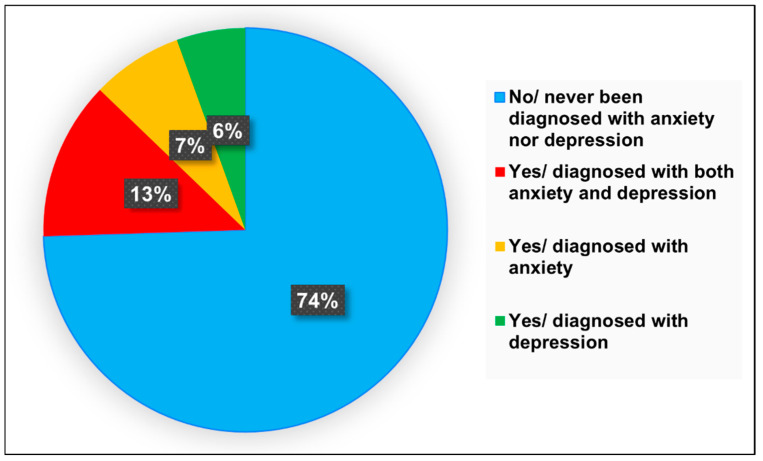
Pie chart illustrating the proportion of participants diagnosed with anxiety and depression.

A large proportion of students (74.5%) reported that they had never been formally diagnosed with anxiety or depression. However, self-reported symptoms indicated that 38.8% were experiencing signs of major depressive disorder (MDD), and 46.1% showed signs of generalized anxiety disorder (GAD). The most common symptoms reported were fatigue or loss of energy (70.6%), sleep disturbances (65.7%), and loss of interest in daily activities (65.0%). Notably, 53.8% believed that anxiety or depression negatively impacted their academic performance, and 43.4% reported experiencing suicidal thoughts. While 55.2% had sought psychiatric evaluation or medication management, 20.3% had pursued counseling therapy. The most cited triggers included academic pressure (79.7%), fear of failure (79.7%), difficulty studying (65.0%), and workload (65.0%) (Figure 1) (Table 2).

### 3.3. Anti-Anxiety and Antidepressant Medication Usage and Perceptions

More than half of the participants (56.6%) reported they are not currently using any medications, while 23.8% are using antidepressants, 5.6% are using anti-anxiety medications, and 14.0% are using both. Among current medication users, selective serotonin reuptake inhibitors (SSRIs) were the most commonly used (71.7%). Escitalopram was the most frequently used SSRI (19.5%). Among those medications, most prescriptions were issued by healthcare professionals (62.2%), and 27.3% had been on medication for less than six months.

**Figure 2:**
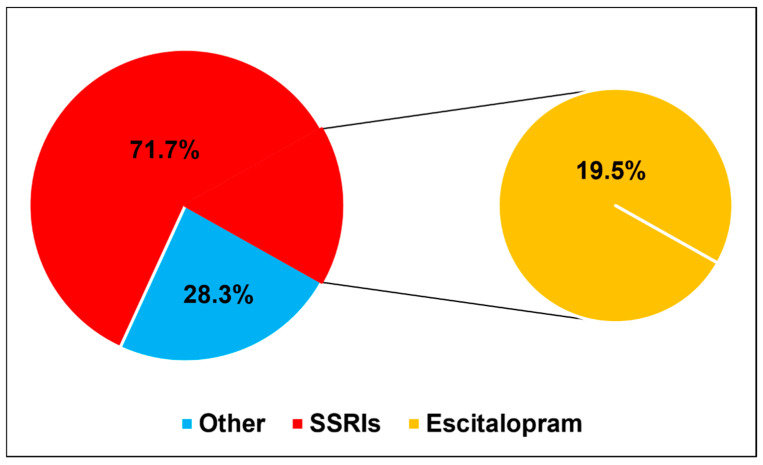
Pie chart demonstrating the percentage of participants using SSRIs and other medications, with a focus on escitalopram as the most commonly used SSRI.

Among ever users, adverse effects were reported by 58.8%, and 39.6% changed medications due to inefficacy or side effects. Almost half (49.0%) discontinued their medications without professional consultation. Despite this, 62.0% of participants felt that the medications improved their academic performance, and 55.6% adhered to their treatment regimens. Although 56.6% reported feeling “doped up” or strange, 76.2% were willing to use them again if needed, and 66.4% reported feeling more relaxed. Furthermore, 74.1% disagreed with using medications only when feeling unwell, 60.8% believed the medications made them feel normal, and 61.5% said they enhanced clarity of thought. A significant majority (77.6%) agreed that these medications can prevent mental breakdowns. (Figure 2) (Table 3).

### 3.4. Associations of Anti-Anxiety and Antidepressant Medication Use with Sociodemographic Data

The analysis revealed that sex and medication use were not significantly related (*p* value = 0.595), with 50.0% of males and 58.7% of females not using medications and 32.4% of males using antidepressants compared with 21.1% of females. Age was also not significantly associated with medication use (*p* value = 0.488), although most users of antidepressants were aged 26–30 years and over 30 years (50.0%). Geographically, participants from the North area had the highest nonuse rate (100%), but this difference was not statistically significant (*p* value = 0.805). No significant associations were found between university attendance and medication use (*p* value = 0.116), although students from Ummul Qura University had the highest nonuse rate (90.0%). Academic year did not show a statistically significant relationship with medication use (*p* value = 0.527), with second-year students (38.5%) being the most antidepressant users and fifth-year students (10.0%) being the most anti-anxiety users. In terms of academic performance (*p* value = 0.368), the majority of students with excellent academic performance did not use anti-anxiety or antidepressant medications (69.7%). Most of the students with poor academic performance were found to be taking antidepressant medications (40.0%) and both anti-anxiety and antidepressant medications (30.0%). Mother’s education level was not significantly associated with medication use (*p* value = 0.496), with the majority of nonusers holding high diplomas (66.7%). Similarly, the father’s education level showed no significant difference (*p* value = 0.241), with the majority of nonusers having a PhD (66.7%) and the uneducated fathers not using anti-anxiety and antidepressant medications (66.7%). Socioeconomic status was not statistically significant (*p* value = 0.093), with lower-class participants showing a greater likelihood of using antidepressant medications (66.7%). Sleep duration was significantly associated with medication use (*p* value = 0.002), as those sleeping more than 10 h per day had a higher rate of antidepressant use (58.3%). Physical activity also showed a significant association (*p* value = 0.014), with those exercising less having a greater likelihood of using both medications. Having a family history of psychiatric disease was also significantly associated with medication use (*p* value = 0.042), with a greater proportion of those with a family history of antidepressant use (26.6%). Smoking, either cigarettes (*p* value = 0.379) or water-pipe (*p* value = 0.756), was not significantly associated with medication use (Table 4).

The regression analysis revealed that students who sleep 8–10 h per day are 7.27 times more likely to use anti-anxiety and/or antidepressant medications than those who sleep 6 h or less (*p* value = 0.019). In comparison, those who slept more than 10 h were 33 times more likely to use these medications (*p* value = 0.001). Compared with not engaging in physical activity, participating in physical activity 1–2 times/week was linked to 3.49 times greater odds of using anti-anxiety or antidepressant medications (*p* value = 0.017), whereas exercising 3–4 times per week or more did not have significant effects. A family history of psychiatric disease was not significantly associated with medication use (Table 5).

## 4. Discussion

This study presents valuable insights into the use of antidepressants and anti-anxiety medications among medical students in Saudi Arabia, indicating important patterns and consequences for mental and educational well-being. The findings reveal a concerning prevalence of mental health challenges within this population, with 38.8% of participants reporting symptoms consistent with major depressive disorder (MDD) and 46.1% experiencing generalized anxiety disorder (GAD). These rates are consistent with other studies that have shown that medical students’ stress, depression, and anxiety levels are far more pronounced than those of students in different professions because of the intense pressures associated with medical education and the competitive culture that contributes to mental health issues [27,28]. In contrast, a systematic review conducted from 2000–2015 reported a lower prevalence rate of 11% for depression and 7% for anxiety disorders among medical students in Asia [29]. The current study addresses a specific gap in Saudi literature by quantifying antidepressant and anti-anxiety medication usage and examining associations with sociodemographic and lifestyle factors, an area previously underexplored in national research.

The results revealed that slightly less than one-fourth of the students (23.8%) were currently taking antidepressants, and 5.6% were taking anti-anxiety medications. Interestingly, a substantial proportion (14.0%) of the students used a combination of both kinds of medications. This widespread use underlines the urgent need for medical schools to address mental health proactively and to foster an environment that encourages students to seek help without stigma [30,31]. Rich et al. reported that the stigma surrounding mental health and fear of career damage can prevent students from following needed treatment, exacerbating their conditions [32]. The study also emphasized that mental health problems strongly affect medical students’ learning and progress in their studies. It is crucial to prioritize addressing the cultural factors within medical education that contribute to these mental health challenges, as well as the barriers preventing students from seeking help. This will create a healthier and more resilient medical academic environment [32]. Additionally, the findings of this study indicate that 71.7% of medical students who were currently using medications were on SSRIs. This finding agrees with other studies, where the prescription of SSRIs is quite common due to their effectiveness in treating anxiety and depression [33,34].

The fact that healthcare professionals prescribed 62.2% of ever-used antidepressants and anti-anxiety medications highlights the importance of professional guidance in treating mental health disorders. However, the relatively high percentage (27.3%) of participants who have ever been on medications, taking these medications for less than six months, is a cause for concern regarding the adequacy of follow-up care and monitoring. Previous research has demonstrated that poor follow-up often leads to noncompliance with medication and, thus, poor treatment outcomes, especially in populations under stress, such as medical students [35].

The side effects reported by 58.8% and 39.6% of users who changed medications for inefficiency or adverse reactions, respectively, are consistent with findings from other studies [36,37]. For example, a study related to the use of escitalopram for the treatment of functional gastrointestinal disorders revealed that side effects, including symptoms of fatigue and dizziness, were frequent in many patients [38]. Furthermore, the fact that 49.0% of discontinuations were without health professional advice is even more worrying, as it might be an indication of poor support and education about adherence to prescribed treatments. This behavior has been reported in other populations, in which patients often discontinue medications either because of side effects or because of perceived ineffectiveness, without professional advice [35].

Despite these challenges, most participants (62.0%) felt that their medications had a positive effect on academic performance. The benefit perception here is similar to what has been reported in other studies, where patients described improvements in functionality and quality of life while on antidepressants [39,40]. However, the complex relationship is further elaborated by the fact that 56.6% of the participants acknowledged that medications made them feel “doped up” or strange. The duality of feeling improved versus impaired is a common issue, with benefits often weighed against side effects [41,42]. Interestingly, 76.2% said they would continue taking medicines if necessary, and 66.4% believed these medications induced relaxation. Overall, the evidence suggests that antidepressant treatment can positively impact functioning and quality of life in medical patients, but individual responses may vary.

Our analysis revealed that the academic pressure of medical students, as well as personal factors such as sleep patterns and physical activity levels, have a substantial effect on medication use. For example, students who slept more than 10 h per day and those who were less physically active demonstrated higher antidepressant usage rates. This finding suggests that the quality of sleep is more important than sleep duration in shaping good mental health. This finding is supported by findings from research studies that consistently established that lifestyle factors, especially sleep quality and physical health, are powerful predictors of mental well-being among medical students. Poor sleep quality is prevalent in 62–63% of individuals and is associated with high levels and rates of depression, anxiety, and stress [43,44]. Unhealthy lifestyles, including poor diet and low physical activity, are strongly correlated with psychological distress [45,46]. Sleep disturbances and autonomic dysfunction are linked to diminished well-being and impaired functioning [47]. Therefore, encouraging healthy lifestyles, including improved sleep hygiene and physical activity, is crucial for enhancing medical students’ mental health and overall well-being [48]. Addressing these factors is essential for supporting students’ academic performance for future roles as healthcare providers.

In addition, the study revealed that a family history of psychiatric disorders increases the probability of students taking antidepressants, thus indicating the influence of genetic and family factors on their mental health. This result is in accordance with various studies that reported the associations of a family history of psychiatric disorders with an increased risk for depression and the use of antidepressants among medical students [49,50]. Moreover, Kadhim et al. reported that medical students have a greater tendency to use antipsychotic medications, whether they are prescribed by physicians or through self-medication, which means that the engagement and support of college students, especially in medical courses, should be strengthened for better academic experiences [51]. Fasanella et al. reported that the prevalence of psychotropic drug use among medical students is approximately 30.4%, and antidepressants are the most widely used psychotropic drugs [33]. Substance abuse, the loss of a relative, and a competitive academic environment are some of the factors that lead to psychological distress [49,50,51].

Interestingly, the results do not show any significant associations between gender and medication use; thus, both male and female students are similarly vulnerable to the challenges of mental health. In contrast, several studies have reported that female students consistently report higher levels of stress, depression, and anxiety than male students do [52,53,54,55]. Women also use more antidepressants and anxiolytics than men do [53,54]. Therefore, the lack of a significant association in this study may reflect a shift in how male and female students perceive and respond to mental health challenges within a medical school context. It is possible that societal stigma around mental health issues is decreasing, leading both genders to seek help more equally. Moreover, high levels of stress in the course of medical education can create a standard environment that increases psychopathology across genders and leads to similar patterns of use of medications. On the other hand, two studies from Germany showed similar or comparable trends in psychotropic medication use among the two genders in clinical practices and inpatient settings, which may indirectly provide some support for our current findings [56,57]. Maybe, studies from controlled settings with minor sociodemographic variations and equal access to care tend to show fewer variable patterns of use compared to studies from general populations and samples with variable backgrounds. In Saudi Arabia, general health facilities and medical services are widely available and accessible to everyone in the community, resulting in fewer inequalities between people and, consequently, fewer gender disparities.

The findings emphasize the importance of targeted mental health interventions in Saudi medical schools. Institutions should implement proactive strategies such as confidential counseling services, mental health awareness campaigns, peer-support programs, and early screening for depression and anxiety. Moreover, medical curricula should incorporate wellness education, stress management workshops, and guidance on lifestyle modifications, including exercise and sleep hygiene. These evidence-based strategies can help foster a healthier educational environment and enhance students’ academic and emotional outcomes.

This study has several limitations that should be addressed. First, cross-sectional analysis is designed to define data information at a single time point. As a result, the ability to identify the underlying relationships between antidepressant/anti-anxiety medication and mental health outcomes may be limited. Additionally, reliance on self-report questionnaires is liable to bias, as respondents may underreport or overreport their mental health condition. In addition, no clinical assessments were used to confirm the mental health status of the participants. Convenience sampling may not accurately represent the entire population of medical students in the Saudi Arabian region, which could potentially affect the extrapolation of the results. Due to the non-probability nature, the sample may be biased, with students who suffer from more severe mental health concerns being more likely to participate in the study. The examination also did not investigate the details of medication control and did not consider other possible confounding factors, such as personality characteristics and other potentially relevant stressful life events in mental health. Furthermore, the study did not assess the role of institutional or academic pressures directly, which may be important contextual variables influencing mental health and medication usage, something that can be addressed in future research. Finally, participants’ attitudes toward the influence of antidepressants and anti-anxiety drugs on their grades are self-evaluations and may also bias their assessments. Overcoming these constraints in future studies may better elucidate mental health issues and medication usage in medical students. Future studies should consider longitudinal designs, diverse sampling strategies, and inclusion of clinical assessments to provide more comprehensive insights into the mental health landscape of medical students.

## 5. Conclusions

This study revealed that the prevalence of mental disorders was significantly high among Saudi medical students, where 38.8% manifested symptoms of MDD and 46.1% GAD. Many of them are using antidepressants and anti-anxiety medications, mostly without adequate professional support or follow-up. A notable concern was the high rate of self-medication and discontinuation of treatment without medical consultation, indicating gaps in mental health education and clinical oversight.

The majority reported positive effects on their academic performance, whereas an appreciable portion experienced side effects and stopped treatment without consulting their health providers. The findings also showed that lifestyle factors, such as sleep and exercise, modulate the use of medications, thus suggesting the promise of improved mental health. These findings underscore the need for educational policies to include proactive mental health support systems and structured follow-up for students using psychotropic medications. The results emphasize the necessity of medical schools creating an environment of support that not only promotes mental health concerns but also offers resources. Such measures ensure student wellness and academic well-being and contribute to maladaptive future health service provisions. Future research should include longitudinal designs to monitor changes in medication use and mental health outcomes over time, as well as qualitative studies that explore the personal experiences, challenges, and decision-making processes of medical students regarding mental health and treatment. In addition, medical schools should foster an environment that encourages medical students to use these drugs responsibly and, in turn, develop specific guidelines to enhance the medical curriculum. Furthermore, it is crucial that medical centers offer appropriate teaching programs related to antidepressant and anti-anxiety drug use and healthy lifestyle practices, and that relevant data on the prevalence of use and understanding of surrounding factors and complications are available.

## Figures and Tables

**Table 1:** Sociodemographic data (*n* = 561).

Parameter	Characteristic	Frequency *n* (%)
**Gender**	Male	160 (28.5)
	Female	401 (71.5)
**Age**	18–20	190 (33.9)
	21–25	352 (62.7)
	26–30	17 (3.0)
	Over 30 Years old	2 (0.4)
**Location**	North area	14 (2.5)
	South area	71 (12.7)
	East area	49 (8.7)
	West area	239 (42.6)
	Center “Middle” area	188 (33.5)
**University**	Taif University	46 (8.2)
	King Abdulaziz University	35 (6.2)
	University of Jeddah	20 (3.6)
	AlRayan Colleges	7 (1.2)
	Al-Baha University	74 (13.2)
	Ummul Qura University—Makkah	111 (19.8)
	King Saud bin Abdulaziz University for Health Sciences (KSAU-HS)—Riyadh	113 (20.1)
	King Saud bin Abdulaziz University for Health Sciences—Jeddah	23 (4.1)
	Other	132 (23.5)
**Academic year**	First year	125 (22.3)
	Second year	53 (9.4)
	Third year	69 (12.3)
	Fourth year	83 (14.8)
	Fifth year	92 (16.4)
	Sixth year	45 (8.0)
	Intern	94 (16.8)
**Rate of academic year performance**	Poor	20 (3.6)
	Fair	33 (5.9)
	Good	146 (26.0)
	Very Good	189 (33.7)
	Excellent	173 (30.8)
**Educational level of mother**	Not educated	22 (3.9)
	School	109 (19.4)
	High diploma	48 (8.6)
	Bachelors	301 (53.7)
	Master	55 (9.8)
	PhD	26 (4.6)
**Educational level of father**	Not educated	14 (2.5)
	School	98 (17.5)
	High diploma	55 (9.8)
	Bachelors	243 (43.3)
	Master	75 (13.4)
	PhD	76 (13.5)
**Socioeconomic status**	Upper class	17 (3.0)
	Upper-middle class	201 (35.8)
	Lower-middle class	43 (7.7)
	Middle class	291 (51.9)
	Lower class	9 (1.6)
**How many hours per day do you sleep?**	6 h or less	139 (24.8)
	6–8 h	286 (51.0)
	8–10 h	110 (19.6)
	More than 10 h	26 (4.6)
**How often do you engage in exercise or physical activity per week?**	None	234 (41.7)
	1–2 times per week	194 (34.6)
	3–4 times per week	102 (18.2)
	5 or more times per week	31 (5.5)
**Is there a family history of psychiatric disease?**	No	395 (70.4)
	Yes	166 (29.6)
**Do you smoke cigarettes?**	No	537 (95.7)
	Yes	24 (4.3)
**Do you smoke a water-pipe (Shisha)?**	No	529 (94.3)
	Sometimes	26 (4.6)
	Yes	6 (1.1)

**Table 2:** Understanding anxiety and depression among medical students.

Parameter	Category	Frequency *n* (%)
**What type of depression do you have?**	Unsure	31 (23.1)
	Bipolar Disorder (Manic Depression)	5 (3.7)
	Major Depressive Disorder (MDD)	52 (38.8)
	Situational Depression (Triggered by specific life events)	7 (5.2)
	Borderline personality disorder with histrionic personality traits	2 (1.5)
	Psychotic Depression	2 (1.5)
	Atypical Depression	2 (1.5)
	None of the above.	22 (16.4)
	Other	11 (8.2)
**What type of anxiety do you have?**	Unsure	26 (20.3)
	Generalized Anxiety Disorder (GAD)	59 (46.1)
	Social Anxiety Disorder (Social Phobia)	6 (4.7)
	Obsessive Compulsive Disorder (OCD)	8 (6.3)
	Panic Disorder	12 (9.4)
	Post-Traumatic Stress Disorder (PTSD)	3 (2.3)
	None of above	11 (8.6)
	Other	3 (2.3)
**What specific symptoms of anxiety or depression are you currently experiencing?**	Persistent feelings of sadness or emptiness	88 (61.5)
	Difficulty sleeping or excessive sleeping	94 (65.7)
	Difficulty concentrating or making decisions	89 (62.2)
	Persistent physical symptoms that do not have a clear medical cause	46 (32.2)
	Loss of interest or pleasure in activities once enjoyed	93 (65.0)
	Changes in appetite or weight	77 (53.8)
	Fatigue or loss of energy	101 (70.6)
	Feelings of worthlessness or excessive guilt	72 (50.3)
	Thoughts of death or suicide	55 (38.5)
	Excessive worry or rumination	62 (43.4)
	Muscle tension or physical symptoms of anxiety (e.g., trembling)	54 (37.8)
	Panic attacks or sudden intense fear	51 (35.7)
	Palpitations (heart palpitations or irregular heartbeat)	55 (38.5)
	Restlessness or irritability	74 (51.7)
	Racing thoughts or mind going blank	60 (42.0)
	Sweating (excessive sweating, especially without physical exertion)	35 (24.5)
**Do you feel that the anxiety or depression has influenced your academic performance?**	No impact	14 (9.8)
	Slightly impacted	16 (11.2)
	Moderately impacted	36 (25.2)
	Significantly impacted	77 (53.8)
**Have you ever experienced suicidal thoughts or attempted suicide?**	No	60 (42.0)
	Yes, suicidal thoughts	62 (43.4)
	Yes, attempted suicide	21 (14.7)
**Have you sought help or support for your mental health?**	No/not considering seeking support	4 (2.8)
	No/considering seeking support	16 (11.2)
	Psychiatric evaluation/medication management	79 (55.2)
	Counseling therapy	29 (20.3)
	Friends or colleagues support	3 (2.1)
	Family support	12 (8.4)
**What do you believe are the primary reasons for anxiety or depression among medical students?**	Difficulty of study	93 (65.0)
	High workload	93 (65.0)
	Sleep deprivation	80 (55.9)
	Pressure to perform well academically	114 (79.7)
	Pressure to excel in clinical rotations	63 (44.1)
	Balancing personal and professional life	80 (55.9)
	Financial concerns or debt	45 (31.5)
	Isolation or lack of social support	77 (53.8)
	Perfectionism	91 (63.6)
	Witnessing or experiencing traumatic events in clinical settings	25 (17.5)
	Fear of failure	114 (79.7)
	Lack of exercise or physical activity	53 (37.1)
	Family issues	78 (54.5)
	Relationship issues	56 (39.2)
	Concerns about future career prospects	78 (54.5)
	Discrimination or stigma within the medical community	39 (27.3)
	Substance abuse or addiction	13 (9.1)
	Genetic predisposition or family history of depression	54 (37.8)
	Chronic diseases (e.g., diabetes, autoimmune disorders)	25 (17.5)

**Table 3:** Anti-anxiety and antidepressant medication usage and perceptions.

Parameter	Category	Frequency *n* (%)
**Are you currently using anti-anxiety and/or antidepressant medications?**	No	81 (56.6)
	Yes/currently using antidepressant medications	34 (23.8)
	Yes/currently using anti-anxiety medications	8 (5.6)
	Yes/currently using both antidepressant and anti-anxiety medications	20 (14.0)
**If yes, what is the name or class of the anti-anxiety and/or antidepressant medications you are currently using?**	SSRIs	43 (71.7)
	Other	17 (28.3)
**How do you obtain anti-anxiety and/or antidepressant medications?**	No medication used	45 (31.5)
	From a friend or family member	4 (2.8)
	Bought over-the-counter	5 (3.5)
	Prescribed by a healthcare professional	89 (62.2)
**How long have you been using anti-anxiety and/or antidepressants?**	No medication used	45 (31.5)
	Less than 6 months	39 (27.3)
	6 months to 1 year	21 (14.7)
	1–2 years	16 (11.2)
	More than 2 years	22 (15.4)
**Have you experienced any side effects from anti-anxiety and/or antidepressant medications?**	No	42 (41.2)
	Yes	60 (58.8)
**Have you ever changed anti-anxiety and/or antidepressant medications due to inefficiency or side effects?**	No	61 (60.4)
	Yes	40 (39.6)
**Have you ever stopped taking anti-anxiety and/or antidepressant medication without consulting a healthcare professional?**	No	52 (51.0)
	Yes	50 (49.0)
**Do you feel that anti-anxiety and/or antidepressant medications have influenced your academic performance?**	No	38 (38.0)
	Yes	62 (62.0)
**Have you been compliant with your anti-anxiety and/or antidepressant medications regimen?**	No	44 (44.4)
	Yes	55 (55.6)
**The positive effects of anti-anxiety and/or antidepressants outweigh the negative effects.**	Disagree	38 (26.6)
	Agree	105 (73.4)
**Using anti-anxiety and/or antidepressants makes individuals feel strange or “doped up.”**	Disagree	62 (43.4)
	Agree	81 (56.6)
**If require anti-anxiety and/or antidepressants, I would take them willingly.**	Disagree	34 (23.8)
	Agree	109 (76.2)
**Antidepressants induce feelings of relaxation.**	Disagree	48 (33.6)
	Agree	95 (66.4)
**Anti-anxiety and/or antidepressants cause feelings of tiredness and sluggishness.**	Disagree	62 (43.4)
	Agree	81 (56.6)
**Anti-anxiety and/or antidepressants are only taken when feeling unwell.**	Disagree	106 (74.1)
	Agree	37 (25.9)
**Anti-anxiety and/or antidepressants make individuals feel normal.**	Disagree	56 (39.2)
	Agree	87 (60.8)
**It feels unnatural for one’s mind and body to be controlled by anti-anxiety and/or antidepressants.**	Disagree	75 (52.4)
	Agree	68 (47.6)
**Those who take anti-anxiety and/or antidepressants experience clearer thoughts.**	Disagree	55 (38.5)
	Agree	88 (61.5)
**Anti-anxiety and/or antidepressants can prevent individuals from experiencing a mental breakdown.**	Disagree	32 (22.4)
	Agree	111 (77.6)

**Table 4:** Association of anti-anxiety and antidepressant medication use with sociodemographic data.

Parameter	Category	No Frequency *n* (%)	Yes/Currently Using Antidepressant Medications Frequency *n* (%)	Yes/Currently Using Anti-anxiety Medications Frequency *n* (%)	Yes/Currently Using Both Antidepressant and Anti-anxiety Medications Frequency *n* (%)	*p* Value
**Gender**	Male	17 (50.0)	11 (32.4)	2 (5.9)	4 (11.8)	0.595
	Female	64 (58.7)	23 (21.1)	6 (5.5)	16 (14.7)	
**Age**	18–20	20 (71.4)	6 (21.4)	0 (0.0)	2 (7.1)	0.488
	21–25	58 (54.2)	24 (22.4)	8 (7.5)	17 (15.9)	
	26–30	2 (33.3)	3 (50.0)	0 (0.0)	1 (16.7)	
	Over 30 Years old	1 (50.0)	1 (50.0)	0 (0.0)	0 (0.0)	
**Location**	North area	4 (100.0)	0 (0.0)	0 (0.0)	0 (0.0)	0.805
	South area	15 (65.2)	5 (21.7)	1 (4.3)	2 (8.7)	
	East area	8 (50.0)	5 (31.3)	1 (6.3)	2 (12.5)	
	West area	24 (49.0)	15 (30.6)	2 (4.1)	8 (16.3)	
	Center “Middle” area	30 (58.8)	9 (17.6)	4 (7.8)	8 (15.7)	
**University**	Taif University	6 (40.0)	6 (40.0)	1 (6.7)	2 (13.3)	0.116
	King Abdulaziz University	6 (40.0)	7 (46.7)	1 (6.7)	1 (6.7)	
	University of Jeddah	3 (60.0)	1 (20.0)	0 (0.0)	1 (20.0)	
	Al-Baha University	17 (73.9)	2 (8.7)	1 (4.3)	3 (13.0)	
	Ummul Qura University—Makkah	9 (90.0)	1 (10.0)	0 (0.0)	0 (0.0)	
	King Saud bin Abdulaziz University for Health Sciences (KSAU-HS)—Riyadh	17 (56.7)	6 (20.0)	4 (13.3)	3 (10.0)	
	King Saud bin Abdulaziz University For Health Sciences—Jeddah	1 (16.7)	3 (50.0)	1 (16.7)	1 (16.7)	
	Other	22 (56.4)	8 (20.5)	0 (0.0)	9 (23.1)	
**Academic year**	First year	9 (69.2)	3 (23.1)	0 (0.0)	1 (7.7)	0.527
	Second year	6 (46.2)	5 (38.5)	0 (0.0)	2 (15.4)	
	Third year	9 (47.4)	5 (26.3)	1 (5.3)	4 (21.1)	
	Fourth year	15 (75.0)	1 (5.0)	0 (0.0)	4 (20.0)	
	Fifth year	15 (50.0)	6 (20.0)	3 (10.0)	6 (20.0)	
	Sixth year	8 (53.3)	4 (26.7)	1 (6.7)	2 (13.3)	
	Intern	19 (57.6)	10 (30.3)	3 (9.1)	1 (3.0)	
**Academic performance**	Poor	3 (30.0)	4 (40.0)	0 (0.0)	3 (30.0)	0.368
	Fair	10 (58.8)	5 (29.4)	0 (0.0)	2 (11.8)	
	Good	19 (48.7)	12 (30.8)	4 (10.3)	4 (10.3)	
	Very Good	26 (59.1)	8 (18.2)	2 (4.5)	8 (18.2)	
	Excellent	23 (69.7)	5 (15.2)	2 (6.1)	3 (9.1)	
**Educational level of mother**	Not educated	3 (42.9)	3 (42.9)	0 (0.0)	1 (14.3)	0.496
	School	16 (50.0)	9 (28.1)	1 (3.1)	6 (18.8)	
	High diploma	6 (66.7)	2 (22.2)	0 (0.0)	1 (11.1)	
	Bachelors	45 (61.6)	14 (19.2)	7 (9.6)	7 (9.6)	
	Master	9 (60.0)	4 (26.7)	0 (0.0)	2 (13.3)	
	PhD	2 (28.6)	2 (28.6)	0 (0.0)	3 (42.9)	
**Educational level of father**	Not educated	4 (66.7)	2 (33.3)	0 (0.0)	0 (0.0)	0.241
	School	8 (34.8)	8 (34.8)	1 (4.3)	6 (26.1)	
	High diploma	6 (37.5)	3 (18.8)	3 (18.8)	4 (25.0)	
	Bachelors	40 (64.5)	14 (22.6)	2 (3.2)	6 (9.7)	
	Master	7 (58.3)	2 (16.7)	1 (8.3)	2 (16.7)	
	PhD	16 (66.7)	5 (20.8)	1 (4.2)	2 (8.3)	
**Socioeconomic status**	Upper class	1 (50.0)	1 (50.0)	0 (0.0)	0 (0.0)	0.093
	Upper-middle class	33 (62.3)	9 (17.0)	4 (7.5)	7 (13.2)	
	Lower-middle class	5 (38.5)	2 (15.4)	0 (0.0)	6 (46.2)	
	Middle class	41 (56.9)	20 (27.8)	4 (5.6)	7 (9.7)	
	Lower class	1 (33.3)	2 (66.7)	0 (0.0)	0 (0.0)	
**How many hours per day do you sleep?**	6 h or less	19 (70.4)	2 (7.4)	1 (3.7)	5 (18.5)	0.002
	6–8 h	47 (67.1)	14 (20.0)	3 (4.3)	6 (8.6)	
	8–10 h	15 (44.1)	11 (32.4)	3 (8.8)	5 (14.7)	
	More than 10 h	0 (0.0)	7 (58.3)	1 (8.3)	4 (33.3)	
**How often do you engage in exercise or physical activity per week?**	None	31 (48.4)	13 (20.3)	5 (7.8)	15 (23.4)	0.014
	1–2 times per week	31 (63.3)	16 (32.7)	0 (0.0)	2 (4.1)	
	3–4 times per week	16 (66.7)	4 (16.7)	3 (12.5)	1 (4.2)	
	5 or more times per week	3 (50.0)	1 (16.7)	0 (0.0)	2 (33.3)	
**Is there a family history of psychiatric disease?**	No	42 (65.6)	13 (20.3)	5 (7.8)	4 (6.3)	0.042
	Yes	39 (49.4)	21 (26.6)	3 (3.8)	16 (20.3)	
**Do you smoke cigarettes?**	No	76 (58.0)	29 (22.1)	8 (6.1)	18 (13.7)	0.379
	Yes	5 (41.7)	5 (41.7)	0 (0.0)	2 (16.7)	
**Do you smoke water-pipe (Shisha)?**	No	75 (57.3)	29 (22.1)	8 (6.1)	19 (14.5)	0.756
	Sometimes	4 (44.4)	4 (44.4)	0 (0.0)	1 (11.1)	
	Yes	2 (66.7)	1 (33.3)	0 (0.0)	0 (0.0)	

**Table 5:** Regression analysis of antidepressant medication usage with statistically significant sociodemographic data.

Parameter	Category	OR (95% CI)	*p* Value
**How many hours per day do you sleep?**	6 h or less	Ref.	Ref.
	6–8 h	3.477 (0.717–16.874)	0.122
	8–10 h	7.266 (1.378–38.305)	**0.019**
	More than 10 h	33.007 (4.389–248.221)	**0.001**
**How often do you engage in exercise or physical activity per week?**	None	Ref.	Ref.
	1–2 times per week	3.485 (1.250–9.715)	**0.017**
	3–4 times per week	1.596 (0.404–6.307)	0.505
	5 or more times per week	0.861 (0.082–9.067)	0.901
**Is there a family history of psychiatric disease?**	No	Ref.	Ref.
	Yes	1.076 (0.451–2.567)	0.870

OR: odd ratio; CI: confidence interval; Ref: reference category, *p* values in bold indicate statistically significant findings.

## Data Availability

Data available on request from the corresponding author owing to privacy/ethical restrictions.

## Abbreviations

**KSAU-HS:** King Saud bin Abdulaziz University for Health Sciences; **MDD:** Major depressive disorder; **GAD:** Generalized anxiety disorder; **OCD:** Obsessive compulsive disorder; **PTSD:** Post-traumatic stress disorder; **PhD:** Doctor of Philosophy; **SSRIs:** Selective serotonin reuptake inhibitors; **IBM SPSS:** Statistical package for the social sciences; **OR:** Odds ratio; **CI:** Confidence interval; **Ref:** Reference category; **TU:** Taif University.
